# Inhibition of LPS-Induced Inflammatory Response of Oral Mesenchymal Stem Cells in the Presence of Galectin-3

**DOI:** 10.3390/biomedicines11061519

**Published:** 2023-05-24

**Authors:** Alessia Paganelli, Francesca Diomede, Guya Diletta Marconi, Jacopo Pizzicannella, Thangavelu Soundara Rajan, Oriana Trubiani, Roberto Paganelli

**Affiliations:** 1 PhD Course in Clinical and Experimental Medicine, Department of Biomedical, Metabolic and Neural Sciences, University of Modena and Reggio Emilia, 41124 Modena, Italy; alessia.paganelli@gmail.com; 2 Department of Innovative Technologies in Medicine and Dentistry, University “G. d’Annunzio” Chieti-Pescara, 66100 Chieti, Italy; francesca.diomede@unich.it; 3 Department of Engineering and Geology, University “G. d’Annunzio” Chieti-Pescara, Viale Pindaro, 42, 65127 Pescara, Italy; jacopo.pizzicannella@unich.it; 4 Research and Development Unit, Theertha Biopharma Private Limited, KIADB, Industrial Area, Bommasandra, Jigani Link Road, Bangalore 560105, India; soundararajan@theerthabiopharma.com; 5 Saint Camillus International University of Health and Medical Sciences (UniCamillus), 00131 Rome, Italy; roberto.paganelli2@gmail.com

**Keywords:** GAL-3, galectin, inflammation, LPS, mesenchymal stem cell, TLR, NFκB

## Abstract

Galectin-3 (GAL-3) is a beta-galactoside binding lectin produced by mesenchymal stem cells (MSCs) and other cell sources under inflammatory conditions. Several studies have reported that GAL-3 exerts an anti-inflammatory action, regulated by its natural ligand GAL-3 BP. In the present study, we aimed to assess the GAL-3 mediated regulation of the MSC function in an LPS-induced inflammation setting. Human gingival mesenchymal stem cells (hGMSCs) were stimulated in vitro with LPSs; the expression of TLR4, NFκB p65, MyD88 and NALP3 were assessed in the hGMSCs via immunofluorescence imaging using confocal microscopy, Western blot assay, and RT-PCR before and after the addition of GAL-3, both alone and with the addition of its inhibitors. LPSs stimulated the expression of TLR4, NFκB p65, MyD88 and NALP3 in hGMSCs, which was inhibited by GAL-3. The addition of either GAL3-BP or the antibody to GAL-3 were able to revert the GAL-3-mediated effects, restoring the expression of TLR4, NFκB p65, MyD88 and NALP3. GAL-3 induces the downregulation of the LPS-induced inflammatory program in MSCs.

## 1. Introduction

Mesenchymal stromal cells (MSCs) are a peculiar subtype of cells of mesenchymal derivation [1]. MSCs were first recognized and identified as multipotent cells in the bone marrow and were defined by the presence of specific characterizing parameters. In 2006, the Mesenchymal and Tissue Stem Cell Committee of the International Society for Cellular Therapy proposed three minimal properties for defining human MSCs: plastic adherence, multi-lineage differentiation (through chondrocyte, osteoblasts, and adipocyte differentiation assays), and the expression of specific markers (CD105, CD73, and CD90) [2]. Recently, other MSC surface molecules have been identified, including STRO-1, CD106, and CD146 [3,4]. After their initial characterization, other MSC sources have been identified so far [5,6]. Possible MSC sources encompass umbilical-cord, amniotic-fluid, subcutis, dental tissue, skeletal muscle, synovium, liver, lungs, tendons, placenta, dermis, and breast milk [7,8,9,10,11,12]. Beyond the possible slight differences based on their specific origin, all MSC subpopulations share self-renewal capabilities, multipotency and immunomodulatory properties [1,13]. In fact, not only—as stem cells—do MSCs have clonogenic potential, but they are also endowed with the ability to exert regulatory functions on the environment, particularly in areas of inflammation. The first clue to such properties was found when they were cocultured in vitro with IFN-γ and peripheral blood mononuclear cells [14,15]. Previous findings, in particular for periodontal ligament stem cells (PDLMSCs), showed that prolonged exposure to IFN-γ induced the increased expression of hepatocyte growth factor, indoleamine 2,3-dioxygenase (IDO), and TGF-β, and the result was the immunosuppression of lymphocyte proliferation after exposure to the activation/mitogenic signal of Concanavalin A [16]. The effect was due to soluble factors produced by the MSCs in reaction to the IFN-γ released by lymphocytes, showing a bidirectional influence of the signals produced in coculture. This study provides the fundamental messages that the immunosuppressive activities of MSCs are independent of the differentiative programs inscribed in the very nature of MSCs, and that the immunomodulating activities of MSCs are induced in response to an inflammatory milieu, and mediated by soluble factors. MSCs can induce T cell energy through either the release of prostaglandin E2 (PGE2) [17], or also through the induction of T regulatory (Tregs) cells [18]. In other studies, the secretome of MSCs was analyzed; the extracellular vesicles released in cultures contained the anti-inflammatory cytokines IL-10 and TGF-β, along with other uncharacterized molecules [19]. However, the PDLMSCs from tissue with periodontitis (inflamed periodontium) exhibited significantly decreased suppressive effects on T cell proliferation, compared with cells from healthy tissue, and this was due to lower Tregs induction [20]. This may represent a problem for the therapeutic attempts of MSCs trials for periodontitis. It also raises the point of the behavior of MSCs in settings of inflammatory processes, which is one of the points addressed by our study. In a series of studies, MSCs have been found to alter antigen-presentation by dendritic cells, affect cytokine production in monocytes/macrophages and CD4+ T helper cells, as well as CD8+ T lymphocytes and natural killer cells cytotoxicity, and control myeloid-derived suppressor cells and Tregs generation and expansion [2,12,21,22,23].

The expression of several factors, including cytokines, chemokines, growth factors, lipids, and immune-regulatory factors both in conditioned media and in extracellular vesicles—which is generally referred to as the secretome—is pivotal to the regenerative and immunoregulatory functions of MSCs [3,24,25].

MSCs also have a specific inflammatory response to bacterial stimuli [26,27]. Lipopolysaccharide (LPS-PG) is the virulence factor of *P. gingivalis*, a pathogen implicated in the pathogenesis of periodontitis, which induces the host immune response via activating innate and acquired immunity [27,28]. GAL-3, in particular, seems to be highly expressed by MSCs under inflammatory conditions [29]. It is both exposed on the cellular membrane surface, and also released in soluble form, although it has not been studied in extracellular vesicles. In fact, the immunomodulatory abilities of MSCs are not constitutive but relatively highly dependent on a holistic niche and the cooperative induction by surrounding cells, mediated by a host of soluble factors, only partially defined. Being plastic cells, MSCs are constantly influenced by the cytokines in the environment, and adapt their phenotype and function accordingly [22,30,31].

GAL-3 is a beta-galactoside binding endogenous lectin with different ligands on different cells; it mediates mainly anti-inflammatory actions, which are regulated by its natural ligand GAL-3 BP [32,33]. GAL-3 BP is produced by many different cell types (including cancer cells) and its expression is upregulated in several physiological and pathological processes (e.g., inflammation, allergy) [34,35,36,37]. In the oral cavity, GAL-3 can act as a recognition receptor for several bacterial species but also for other microorganisms, stimulating neutrophil function and also having microbicidal activity [38]. However, it has both pro- or anti-inflammatory activities depending on various factors, including the microbiota, and it has been implicated in the pathogenesis of periodontitis [38].

While several authors already reported GAL-3 production by MSCs and other cell types under inflammatory conditions [39,40,41], little is known about a possible GAL-3 mediated regulation of MSC function. In an inflammatory environment, many cell types are producing GAL-3 and this affects their responses. T lymphocytes have been shown to produce GAL-3, which influences the activities of other cells; however, many effects are due to membrane-bound GAL-3, and the lack of autologous immunosuppression on T cells has not been satisfactorily addressed until today [42,43]. In the same line of thought, the effects of exogenous GAL-3 on the function of MSCs have not been studied. Both the regenerative, immunomodulating, and proinflammatory pathways of MSCs might be affected by increased amounts of GAL-3. The present work aims to describe the effects of GAL-3 and its natural inhibitors (including GAL-3BP) on the response of gingival derived MSCs to activation by the inflammation inducer LPS-G in order to widen the current knowledge of its effects on the inflammatory program in MSCs.

## 2. Materials and Methods

### 2.1. Ethic Statement

A consent document was signed by the patients registered in the current work. The study design was formerly accepted by the Medical Ethics Committee at the Medical School, “G. d’Annunzio” University, Chieti, Italy (N° 266/17 April 2014). The Department of Innovative Technologies in Medicine and Dentistry and the Laboratory of Stem Cells and Regenerative Medicine are licensed according to the quality standard ISO 9001:2008 (N° 32031/15/S).

### 2.2. Cell Culture Establishment

Human gingival mesenchymal stem cells (hGMSCs) were isolated from the gingival tissues of three patients, in good general health conditions and without oral disease, who underwent a surgical procedure. The gingival tissues were placed in a culture dish, fragmented, washed several times in phosphate-buffered saline solution (PBS, Lonza, Basel, Switzerland) with 5% of gentamicin (Lonza) and transferred into the incubator at 37 °C in a humidified atmosphere of 5% CO_2_ in air with mesenchymal stem cell growth medium-chemically defined (MSCGM-CD, Lonza). The medium was replaced every two days for approximately two weeks before the cells reached 80% confluence and were passaged in culture [26].

### 2.3. Cellular Characterization

MSCs were recognized as spindle-shaped cells in the bone marrow, characterized by their adherence to plastic in standard cultures and the potential for clonogenic proliferation. As already discussed, the Mesenchymal and Tissue Stem Cell Committee of the International Society for Cellular Therapy, in 2006, defined the minimal criteria for MSCs: plastic adherence, capability to differentiate into three differentiated tissue lines (chondrocytic, osteoblastic and adipocytes) in vitro, and the presence of some supposedly specific surface markers, such as CD105, CD73, and CD90 [2]. This definition has been recently revised, and novel more specific surface molecules have been added, such as STRO-1, CD106, and CD146 positives [4]. According to these guidelines, the morphology and phenotype of cells obtained from gingival tissues were evaluated, respectively, using light microscopy (Leica, DMIL, Milan, Italy) and cytofluorimetry, as previously described [44,45]. The cytofluorimetric analysis was performed through Fluorescence-Activated Cell Sorting (FACS) (Calibur, Becton-Dickinson [BD], San Jose, CA, USA). Briefly, cells were added to 0.1% trypsin-EDTA, harvested and suspended in PBS with 1:100 dilution of mouse monoclonal antibodies directed to the following human antigens, either conjugated with fluorescein isothiocyanate (FITC): HLA-DR, CD45, or with phycoerythrin (PE): CD90, CD105, CD73 (all from BD). MSCs express a number of non-specific markers, including CD105, CD73, CD90, being negative for HLA-DR and CD45 [46]. The labeled cells were acquired using flow cytometer and the data were analyzed using FlowJo™ software (TreeStar, Ashland, OR, USA). The gating combinations were one PE- and one FITC-conjugated antibody in two immunofluorescences.

Osteogenic and adipogenic differentiation assays were also performed [47] in order to validate the differentiation capabilities of the cultured hGMSCs obtained.

Human GMSCs were cultured under osteogenic and adipogenic conditions for 21 and 28 days, respectively. For evaluating the development of the mineralized precipitates and lipid droplets, successively to the differentiation part, Alizarin Red S and Adipo Oil Red staining were executed on the undifferentiated and differentiated cells. For Alizarin Red S, the staining cells were washed with PBS, fixed in 10% (*v*/*v*) formaldehyde (Sigma-Aldrich, Milan, Italy) for 30 min, and washed twice with a lot of distilled water (dH_2_O) prior to adding Alizarin Red S 40 mM in water for 20 min at room temperature. Afterward, the cells were incubated under gentle shaking, and the cells were washed with dH_2_O four times for 5 min. For staining quantification, 800 μL of 10% (*v*/*v*) acetic acid was added to each well. Cells were kept for 30 min with shaking, then scraped from the plate, moved to a 1.5 mL vial, and vortexed for 30 s. The acquired suspension, overlaid with 500 mL mineral oil (Sigma-Aldrich, Milan, Italy), was heated to 85 °C for 10 min, then moved to ice for 5 min, cautiously avoiding opening the tubes until fully cooled, and centrifuged at 20,000× *g* for 15 min. In total, 500 μL of the supernatant was placed into a new 1.5 mL vial, and 200 μL of 10% (*v*/*v*) ammonium hydroxide was added (pH 4.1–pH 4.5)

A total of 150 μL of the supernatant gained from the differentiated and undifferentiated hGMSCs was read in triplicate at 405 nm using a spectrophotometer (Synergy HT, BioTek Instruments, Bad Friedrichshall, Germany). For adipogenic staining, the cells were fixed in 10% formalin for 30 min and washed with dH_2_O. Then, the cells were stained with Oil Red O working solution (300 mg of Oil Red O/100 mL of isopropanol) for 5 min and counterstained with hematoxylin. The differentiation into the adipogenic lineage was analyzed using the AdipoRed assay reagent hydrophilic Nile Red fluorescence (Lonza, Basel, Switzerland). Then, the plates were rinsed with PBS and a 140 mL/well of AdipoRed was added; after 10 min, the fluorescence with an excitation at 485 nm and an emission at 572 nm was evaluated with a fluorimeter (Synergy HT, Winooski, VT, USA) [48].

### 2.4. Experimental Study Design

hGMSCs at passage 3 of the cell culture were exposed to different stimuli. The hGMSCs were stimulated with 5 μg/mL of Ultrapure LPSs derived from *Porphyromonas gingivalis* (LPS-PG) (tlrl-ppglps, Invivogen, Toulouse, France). The cells were also treated with Recombinant Human Galectin-3 at 0.25 μg/mL (1154-GA, R&D Systems, Minneapolis, MN, USA), Human Galectin-3 Antibody at 2 μg/mL (GAL-3 Ab) (MAB11541, R&D Systems), and Recombinant Human Galectin-3BP at 0.25 μg/mL (GAL-3 BP) (2226-GAB, R&D Systems).

The following culturing conditions were assessed in the present study:

- hGMSCs alone, used as a negative control (CTRL);
- hGMSCs cultured with GAL-3 0.25 µg/mL for 72 h;
- hGMSCs cultured with GAL-3 0.25 µg/mL + GAL-3 Ab 2 µg/mL for 72 h;
- hGMSCs cultured with GAL-3 0.25 µg/mL + GAL-3 BP 0,25 µg/mL for 72 h;
- hGMSCs cultured with 5 μg/mL of LPS-PG for 72 h;
- hGMSCs cultured with GAL-3 0.25 µg/mL + 5 μg/mL of LPS-PG for 72 h;
- hGMSCs cultured with GAL-3 0.25 µg/mL + GAL-3 Ab 2 µg/mL and 5 μg/mL of LPS-PG for 72 h;
- hGMSCs cultured with GAL-3 0.25 µg/mL + GAL-3 BP 0.25 µg/mL and 5 μg/mL of LPS-PG for 72 h.

### 2.5. Confocal Laser Scanning Microscope (CLSM) Analysis

To perform the Confocal Laser Scanning Microscope analysis, 6.4 × 10^3^/well of hGMSCs were placed in 8-well culture glass slides and treated with different stimuli for 72 h (as mentioned above). Then, the cells were fixed with 4% of paraformaldehyde (PFA) (BioOptica, Milan, Italy) in PBS (0.1 M) (Lonza, Basel, Switzerland) for 1 h at room temperature, washed 3 times with PBS, permeabilized with 0.1% Triton X-100 (BioOptica) in PBS and blocked for 1 h at room temperature with 5% of skimmed milk in PBS. The primary antibodies anti-TLR4 (sc-293072, Santa Cruz Biotechnology, Dallas, TX, USA), anti-MyD88 (sc-74532, Santa Cruz Biotechnology), anti-NFκB p65 (sc-8008, Santa Cruz Biotechnology) and anti-NALP3 (sc-134306, Santa Cruz, Biotechnology) were diluted 1:200 and incubated overnight at 4 °C. Then, the secondary antibody Alexa Fluor 568 red fluorescence-conjugated goat anti-mouse (1:200) (A11031, Invitrogen, Eugene, OR, USA) was added to the samples for 1 h at room temperature, followed by the final incubation with 1:200 of Alexa Fluor 488 phalloidin green fluorescent conjugate (A12379, Invitrogen) and TOPRO (T3605, Invitrogen) mixed together. The images were obtained with a Zeiss LSM800 confocal system (Carl Zeiss, Jena, Germany) [49].

### 2.6. Western Blot Analysis

The hGMSCs were treated with different stimuli for 72 h and then lysated with a RIPA Lysis and Extraction Buffer (Thermo Fisher Scientific, Waltham, MA, USA) and the proteins were quantized using Eppendorf BioSpectrometer^®^ fluorescence (Eppendorf, Hamburg, Germany). The electrophoresis, executed with 50 µg of sample protein lysates, was followed by a proteins transfer on a polyvinylidenfluoride (PVDF) membrane through the SEMI-dry blotting apparatus (Bio-Rad Laboratories Srl, Milan, Italy. After 2 h of blocking with 5% of skimmed milk in PBS + 0.1%Tween-20 (Sigma-Aldrich), the membranes were incubated overnight at 4 °C with the primary antibodies anti-TLR4 (sc-293072, Santa Cruz Biotechnology), anti-MyD88 (sc-74532, Santa Cruz Biotechnology), anti-NFκB p65 (sc-8008, Santa Cruz Biotechnology), anti-Cryopyrin/NALP3/NLRP3 (sc-134306, Santa Cruz, Biotechnology) diluted 1:500, and anti-β-actin (sc-47778, Santa Cruz Biotechnology) diluted 1:750, used as a loading control. The secondary antibody goat anti-mouse, diluted 1:5000 (A90-116P, Bethyl Laboratories Inc., Montgomery, TX, USA), was incubated for 1 h at room temperature; successively, protein expression was analyzed using an Immobilon Crescendo Western HRP substrate (Merk Millipore, Burlington, MA, USA) through the enhanced chemiluminescence exposure process (ECL), with an image documenter Alliance 2.7 (Uvitec, Cambridge, UK). The signals were acquired using ECL enhancement and assessed through UVIband-1D gel analysis (Uvitec). The experiments were performed in triplicate. The results achieved were normalized with β-actin values [50].

### 2.7. Real-Time RT-PCR Analysis

A PureLink RNA Mini Kit (Ambion, Thermo Fisher Scientific, Milan, Italy) was used to extract the total RNA from the samples. Successively, for the RNA quantification, 1 µg of total RNA of each sample was retrotranscribed through M-MLV Reverse Transcriptase (M1302 Sigma-Aldrich). The cDNA samples obtained were used for the Real-Time PCR, executed through the Mastercycler ep real plex real-time PCR system (Eppendorf) according to the protocol reported by Marconi et al. [51]. TaqMan Gene Expression Assays *TLR4* (Hs.PT.58.38700156.g), *RELA* (Hs.PT.58.22880470), MYD88 (Hs.PT.58.40428647.g), *NLRP3* (Hs.PT.58.39303321) and *B2M* (Beta-2 microglobulin) (Hs99999907_m1) and the PrimeTime^®^ Gene Expression Master Mix were all purchased from Tema Ricerca Srl, (Bologna Italy). *B2M* was utilized for the template normalization (Table 1). Expression levels for each gene were achieved in agreement with the 2^−ΔΔCt^ method. RT-PCR was executed in triplicate.

### 2.8. Statistical Analysis

GraphPad 5 (GraphPad, San Diego, CA, USA) software was used to calculate the statistical significance. Specifically, ordinary one-way ANOVA were initially performed, followed by post hoc Tukey’s multiple comparisons analysis. Values of *p* < 0.05 were considered statistically significant. Regarding gene expression analysis, the comparative 2^−ΔΔCt^ method was used to quantify the relative abundance of mRNA and then to determine the relative changes in individual gene expression (relative quantification) [51].

## 3. Results

### 3.1. Human GMSCs Exhibited the Stemness Markers Expression and the Osteogenic and Adipogenic Differentiation Capacity In Vitro

**Figure 1:**
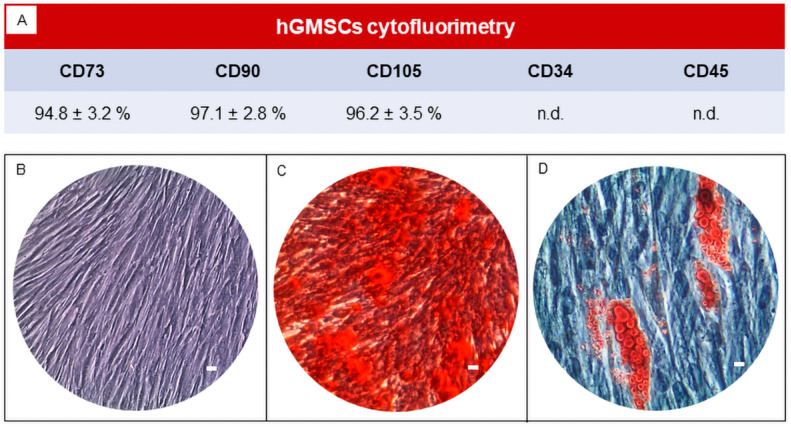
Characterization of hGMSCs. (**A**) Cytofluorimetric value of hGMSCs (n.d.: not detectable). (**B**) hGMSCs cultured in basal medium with a fibroblast-like morphology observed under light microscopy. (**C**) Osteogenic differentiation evaluated using Alizarin S Red staining. (**D**) Adipogenic differentiation evaluated using Oil Red O staining. Scale bars: 20 μm.

First, the cytofluorimetric assay performed on the hGMSCs confirmed that the hGMSCs used in the present study were CD73+, CD90+, CD105+, CD34- and CD45- (Figure 1A), as previously described [44,45]. The human GMSCs showed the ability to adhere on a plastic substrate with a spindle-shape morphology (Figure 1B). The human GMSCs had a multidirectional differentiation potential when maintained in vitro under osteogenic and adipogenic conditions, as demonstrated using Alizarin Red S and Oil red O staining (Figure 1C,D).

### 3.2. GAL-3 Inhibited the Inflammation Pathway in hGMSCs Stimulated with LPS-G

**Figure 2:**
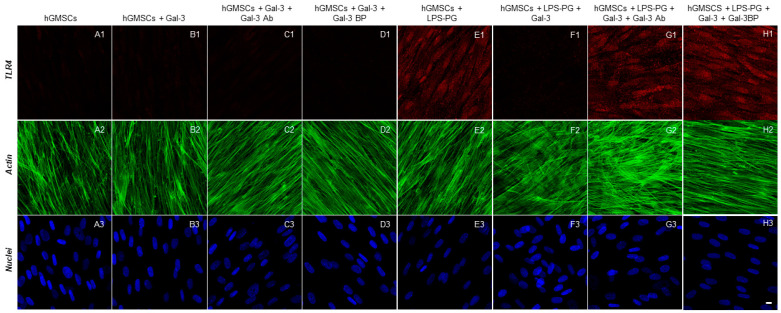
Immunofluorescence analysis of TLR4 expression. (**A1**–**H1**) TLR4 expression stained in red fluorescence. (**A2**–**H2**) Cytoskeleton actin stained in green fluorescence. (**A3**–**H3**) Nuclei stained with TOPRO in blue fluorescence. Scale bar 10 μm.

**Figure 3:**
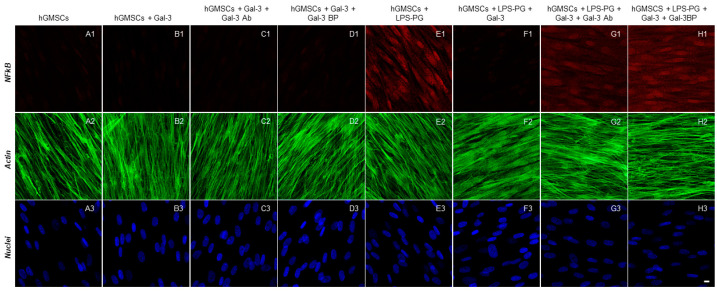
Immunofluorescence analysis of NFκB expression. (**A1**–**H1**) NFκB expression stained in red fluorescence. (**A2**–**H2**) Cytoskeleton actin stained in green fluorescence. (**A3**–**H3**) Nuclei stained with TOPRO in blue fluorescence. Scale bar 10 μm.

**Figure 4:**
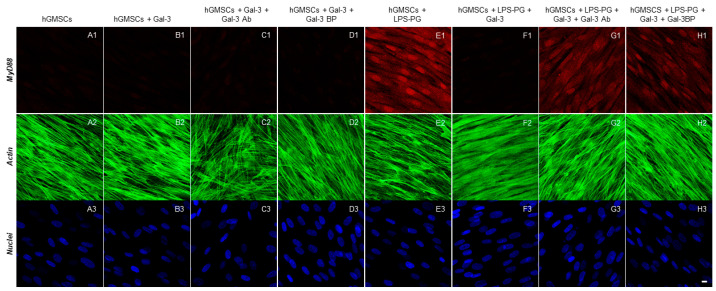
Immunofluorescence analysis of MyD88 expression. (**A1**–**H1**) MyD88 expression stained in red fluorescence. (**A2**–**H2**) Cytoskeleton actin stained in green fluorescence. (**A3**–**H3**) Nuclei stained with TOPRO in blue fluorescence. Scale bar 10 μm.

**Figure 5:**
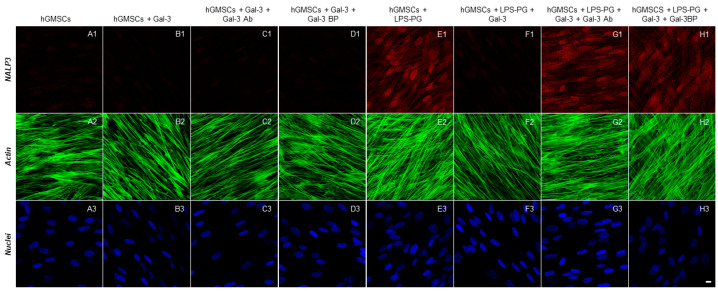
Immunofluorescence analysis of NALP3 expression. (**A1**–**H1**) NALP3 expression stained in red fluorescence. (**A2**–**H2**) Cytoskeleton actin stained in green fluorescence. (**A3**–**H3**) Nuclei stained with TOPRO in blue fluorescence. Scale bar 10 μm.

**Figure 6:**
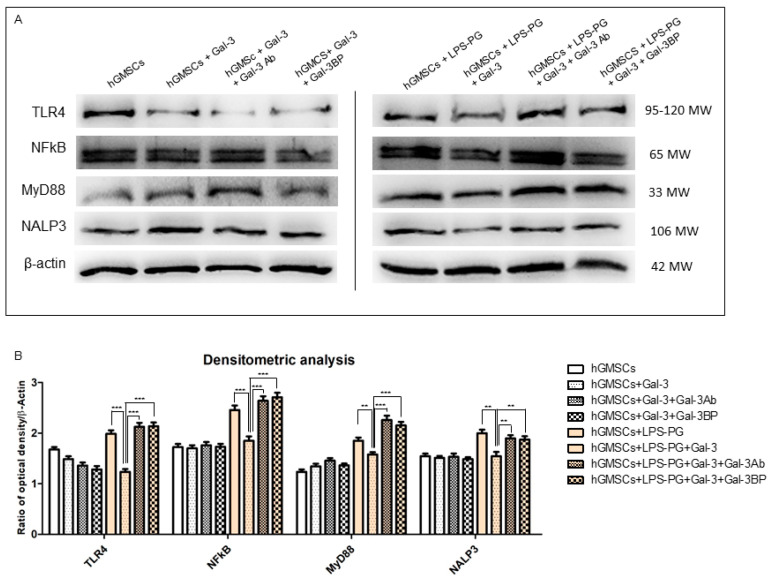
Protein expression analysis through Western Blotting. (**A**) TLR4, NFκB, MyD88 and NALP3 specific band obtained by means of the Western blot experiments. (**B**) Densitometric analysis of TLR4, NFκB, MyD88 and NALP3 normalized using β-actin value. ** *p* < 0.01; *** *p* < 0.001.

**Figure 7:**
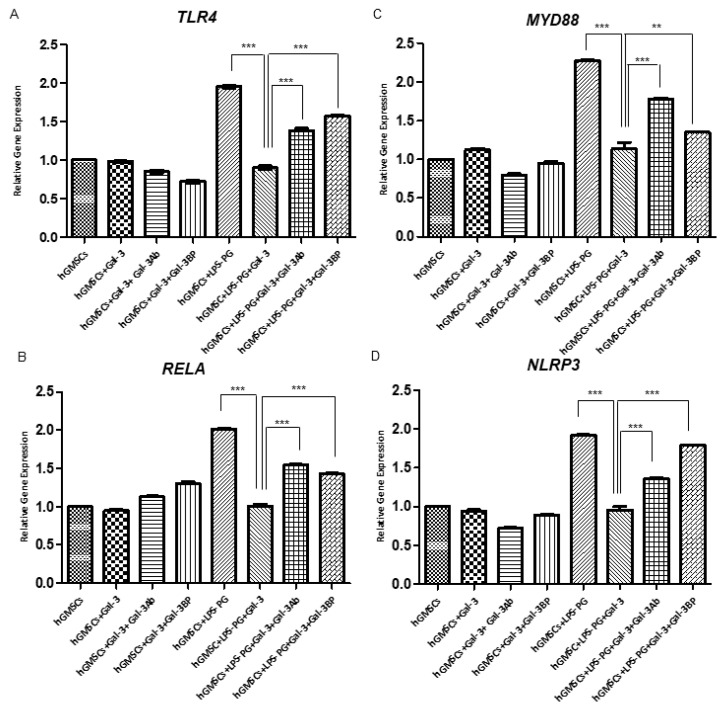
Gene expression analysis. A graph bar reported the RT-PCR results of (**A**) *TLR4*, (**B**) *NFκB*, (**C**) *MyD88*, and (**D**) *NALP3*. All data were normalized using *B2M*. ** *p* < 0.01; *** *p* < 0.001.

The immunofluorescence staining indicated that the inflammatory markers TLR4, NFκB, MyD88 and NALP3 were significantly upregulated by LPS-G stimulation (Figure 2E1, Figure 3E1, Figure 4E1 and Figure 5E1), whereas the GAL-3 treatment reduced the expression of inflammatory proteins (Figure 2F1, Figure 3F1, Figure 4F1 and Figure 5F1). On the other hand, GAL-3 inhibition, obtained either with GAL-3 Ab or GAL-3 BP co-treatment, restored the expression of TLR4, NFκB, MyD88 and NALP3 in the hGMSCs triggered by LPS-PG (Figure 2G1,H1, Figure 3G1,H1, Figure 4G1,H1 and Figure 5 G1,H1). No effect was detected in an unstimulated condition when GAL-3 or GAL-3 and its inhibitors were added (Figure 2B1–D1, Figure 3B1–D1, Figure 4B1–D1 and Figure 5B1–D1). Western blot analyses revealed that TLR4, NFκB, MyD88 and NALP3 were increased in cells stimulated with the LPS-PG and decreased by GAL-3 treatment, such changes being reversed by GAL-3 inhibition using GAL-3 Ab or GAL-3 BP (Figure 6); even some further signal increase could be found compared to the LPS-induced conditions. In addition, in this case, no significant change was observed in the unstimulated cultures. Further validation of these results was also performed on the gene expression using RT-PCR. A significantly increased expression of TLR4, NFκB, MyD88 and NALP3 could be demonstrated in LPS-PG stimulated cells compared to hGMSCs treated with LPS-PG and GAL-3; furthermore, in cells treated with LPS-PG + GAL-3 + GAL-3Ab and LPS-PG + GAL-3 + GAL-3BP, a significant restoration of induction in comparison with the hGMSCs + LPS-PG + GAL-3 could be shown (Figure 7).

## 4. Discussion

The results of our study demonstrate that human GMSCs do express a proinflammatory programmed response to inflammatory stimuli present in the local tissue, such as that due to *P. gingivalis* LPS-PG. The response consists of the activation of both IL-1β dependent and independent pathways, i.e., with TLR4, NALP3, but also NFκB upregulation. The final result would be the secretion of several proinflammatory mediators, but since multiple pathways are activated, other effects cannot be excluded, nor anticipated. This shows that in conditions of inflammation (such as periodontitis), GMSCs might also contribute to the inflammation scenario, with reduced regenerating potential. However, these cells operate in an environment shared with other cells—those mainly resident in the tissue but also immune cells attracted to the scene—which filled with other mediators, including those opposing inflammation-induced activation, such as GAL-3. To complicate this picture, the endogenous inhibitor GAL-3BP is also released in the local inflamed tissue, antagonizing the immunosuppressing action of released GAL-3. Therefore, the delicate balance of activator-inhibitor secretion, such as IL-1RA and IL-1β, or osteoprotegerin for the osteoclast precursors in the RANK-L activation process, needs some timing data, which are notably absent.

Our study confirms that GAL-3 exerts an inhibitory effect on the LPS-induced inflammatory response from MSCs [52]. GAL-3 can also be actively produced by the MSCs themselves, as well as by numerous other cell types, and its secretion is increased in response to LPSs and other inflammatory stimuli. GAL-3 is also displayed on the outer plasma membrane upon inflammatory stimuli [40,41]. The autocrine effect of the secretion of an essential regulatory mediator on the producer cell is not known, but this is also the case for other inhibitory molecules and other cell types producing GAL-3 [16]. GAL-3 shares a basic structure with other galectins, consisting of a carbohydrate-recognition domain (CRD) [53,54]. This reflects the endogenous lectin (sugar-binding) nature of galectins [55]. However, the GAL-3 peculiarity is CRD fusion with a large N-terminal protein-binding domain, providing the reason for its classification as the only chimera-type galectin subgroup [56,57,58]. GAL-3’s function depends not only on the type of producing cell but also on its localization (intracellular or extracellular) [59]. Galectin-3 can bind to two different sites in LPSs (the galactose domain and the lipid A domain) [60], and when this occurs in the extracellular compartment, it alters LPS signaling. Galectin-3 is known to self-associate upon binding to multivalent glycans through its non-lectin domain, and this may lead to multimolecular complexes reaching different cells with different effects: for instance, the addition of GAL-3 to LPSs in a monocyte culture increases the production of IL-1 [52,61,62,63]. Therefore, it is not easy to extend our findings to the effects on cytokine secretion induced by other stimuli or in other cell types. We detected the transcription upregulation of a set of proinflammatory genes, which may translate to inflammatory molecules’ production along the inflammasome and NFκB pathways. Among the known cytokines, IL-6, IL-10, TGF-β are the main products of MSCs [3,64]. Other bioactive mediators are actively produced by MSCs, as MCP-1, IL-11, IL-8, and VEGF [65], and the resulting composition of the secretome cannot be entirely predicted based on such observations.

GAL-3 knockdown in MSCs has been shown to reduce their migratory and proliferative capacities [66,67]. However, MSC-mediated immunomodulation is not always strictly dependent on the local recruitment and homing of MSCs, as shown by previous observations in experimental models of myocarditis [68].

Notably, MSCs are crucial not only in inflammatory conditions but also in anti-tumor immunity [55,69,70,71], although the tumor microenvironment contributes to the shaping of their pro- or anti-tumorigenic function, resulting in the enhancement or suppression of tumor growth [70,72,73,74]. Many tumor cells are among those producing GAL-3 to suppress local immune responses [75,76,77] and this might reduce and compromise natural anti-cancer and therapeutic interventions.

As indicated so far, the action of GAL-3 on MSCs is not univocal [78,79,80]. Even the source of GAL-3 is not always clear, since it is produced by many different cell types, primarily including those driving the inflammatory response. In addition, similar considerations are valid for the counter-regulatory molecule GAL-3BP, which has not been extensively investigated until now [78,79,80]. The knockdown of GAL-3 demonstrated the reduced immunosuppressive effect of MSCs on mixed lymphocyte reactions in vitro and decreased proliferation, survival, and migration in vivo in murine models [66,81,82]. However, GAL-3 absence has not been shown to have a direct impact on the immunophenotype or differentiation potential of MSCs, adding to our hypothesis of a dissociation between these two essential properties of MSCs. In this perspective, GAL-3 may be seen as a regulator of MSC immunosuppressive activity [78], balanced by the presence of GAL-3BP. In order to assess the GAL-3 role(s) on the MSCs’ response to inflammation, we studied a model using LPS-G, a pathogenic driver of *P. gengivalis* inflammation, and its reversal with two different inhibitors, one being a specific monoclonal antibody, and the other a naturally produced antagonist (GAL-3BP). The data obtained indicate that endogenous GAL-3 has very scarce, if any, suppressive activity under our experimental conditions; this is indirectly observed for data on the activation of inflammatory mechanisms. This has been already noted for T lymphocytes, responding well to exogenously added GAL-3, but protected, by an unknown mechanism, from endogenously produced galectins [41]. A similar observation might be made for the endogenous production of GAL-3 in MSCs; however, our data, which are the first to hint to this, seem to indicate that antagonizing endogenous GAL-3 has little or no significant effect on its immunosuppressive activity, as shown and reported for the data in Figure 6 and Figure 7.

Whether the conclusions of GAL-3-mediated action on the LPS-induced inflammation on MSCs could be extended to other inflammatory conditions remains unclear [28,50]. The in vivo production of cytokines and chemokines, as well as the interplay between the different cell types (including lymphocytes, macrophages, dendritic cells, neutrophils…) provide a reason for the more complex biology of the inflammatory response to various exogenous and/or endogenous stimuli [83,84,85,86,87]. This particular problem has not been addressed so far, and the exact composition of the secretome under different experimental conditions remains undetermined.

Future studies on the effects of GAL-3 on other immune cell subpopulations, both residents and those attracted by chemotactic signals, and upon different inflammatory stimuli, are needed in order to not leave these questions unanswered for long.

Increasing numbers of clinical studies are using MSCs for the treatment of many degenerative and inflammatory disorders [1,12,88]. They have also attempted to use them as a therapy for patients with severe COVID-19 [12,88,89]. MSCs have become a powerful new tool for effective immunosuppression, which is devoid of the many adverse effects of conventional immunosuppressant drugs [90]. Dental MSCs share both regenerative and immunoregulatory potentials, which are important for tissue engineering and regenerative medicine [91]. However, there are no comprehensive studies on their interactions with different immune cell types, so the mechanisms of their suppressive activity are not well understood.

Several mechanisms have been suggested for the immunoregulatory activity of MSCs, involving both cell contact and soluble mediators [92]. The secreted mediators, represented mainly by growth factors and cytokines, have a homeostatic and survival role, and stimulate growth through paracrine secretion; they are collectively defined as the secretome. The secretome has been demonstrated to account for many of the effects of MSCs, so it has been surmised that its use can equal the cell’s action, escaping the limitations linked to stem cell treatment [93,94]. The secretome also contains extracellular vesicles (EVs) [95]. These released EVs, which include various species, such as exosomes, microparticles, microvesicles, and apoptotic bodies, can be regarded as an extracellular vesicular compartment, which plays a strategic role in the MSC’s paracrine or autocrine biological effects. The EVs contribute to tissue repair, and also participate in the MSC’s interaction with immune cells due to their content of microRNAs, cytokines, chemokines, growth factors, and other molecules. The balanced regulation of these mediators provides finely tuned immunomodulatory effects and prevents excessive tissue fibrosis during repair; also stimulating angiogenesis [96].

Recent studies have centered on the fact that the aging of MSCs may affect their therapeutic efficacy, mainly decreasing their immunosuppressive capacity [97,98]. Senescent MSCs display a lower production of cytokines and chemokines; they have decreased proliferative activity, and their ability to inhibit T cell proliferation is diminished; this is accompanied by a lack of suppression towards B cells, NK cells and macrophages [88,99,100]. Changes in the expression profiles of senescent MSCs have been found, including transcriptomic, proteomic, epigenetic, and noncoding RNAs. Possible rejuvenation strategies have been suggested, ranging from dietary manipulation to the modulation of the microenvironment.

## 5. Conclusions

GAL-3 interferes with the LPS-induced inflammatory program in MSCs. The paracrine action of GAL-3 produced by MSCs under inflammatory conditions may underlie their well-established immunomodulatory and anti-inflammatory role.

## Figures and Tables

**Table 1:** Primer sequences used for real-time PCR reactions.

Gene	Forward Primer Sequence (5′-3′)	Reverse Primer Sequence (5′-3′)
*TLR4*	5′-GAGTATACATTGCTGTTTCCTGTTG-3′	5′-ACCCCATTAAT- TCCAGACACA-3′
*MYD88*	5′-CGGTCTCCTCCA- CATCCT-3′	5′-GCCGGACCCAA-GTACTCA-3′
*RELA*	5′-CGAGCTTGTAGGAAAGGACTG-3′	5′-TGACTGATAGC- CTGCTCCAG-3′
*NLRP3*	5′-GAATGCCTTGG- GAGACTCAG-3′	5′-AGATTCTGATT- AGTGCTGAGTACC-3′
*B2M*	5′-GGACTGGTCTT-TCTATCTCTTGT-3′	5′-ACCTCCATGAT- GCTGCTTAC-3′

## Data Availability

Data are available from the corresponding author upon request.

## Abbreviations

LPS	lipopolysaccharide
TLR	Toll-like receptor
MSC	mesenchymal stromal/stem cell
hGMSC	human gingival MSC
IL	interleukin
IFN	interferon
PDLMSC	periodontal ligament stem cells
